# Cardiac remodeling and dysfunction in childhood obesity: a cardiovascular magnetic resonance study

**DOI:** 10.1186/s12968-016-0247-0

**Published:** 2016-05-11

**Authors:** Linyuan Jing, Cassi M. Binkley, Jonathan D. Suever, Nivedita Umasankar, Christopher M. Haggerty, Jennifer Rich, Gregory J. Wehner, Sean M. Hamlet, David K. Powell, Aurelia Radulescu, H. Lester Kirchner, Frederick H. Epstein, Brandon K. Fornwalt

**Affiliations:** Departments of Pediatrics, University of Kentucky, Lexington, KY USA; Department of Biomedical Engineering, University of Kentucky, Lexington, KY USA; Department of Physiology and Medicine, University of Kentucky, Lexington, KY USA; Department of Electrical Engineering, University of Kentucky, Lexington, KY USA; Institute for Advanced Application, Geisinger Health System, 100 North Academy Avenue, Danville Pennsylvania, PA 17822-4400 USA; Center for Health Research, Geisinger Health System, Danville Pennsylvania, PA USA; Department of Biomedical Engineering, University of Virginia, Charlottesville, VA USA

**Keywords:** Pediatric obesity, Cardiac magnetic resonance, Cardiac remodeling, Cardiac mechanics

## Abstract

**Background:**

Obesity affects nearly one in five children and is associated with increased risk of premature death. Obesity-related heart disease contributes to premature death. We aimed to use cardiovascular magnetic resonance (CMR) to comprehensively characterize the changes in cardiac geometry and function in obese children.

**Methods and results:**

Forty-one obese/overweight (age 12 ± 3 years, 56 % female) and 29 healthy weight children (age 14 ± 3 years, 41 % female) underwent CMR, including both standard cine imaging and displacement encoded imaging, for a complete assessment of left ventricular (LV) structure and function. After adjusting for age, LV mass index was 23 % greater (27 ± 4 g/m^2.7^ vs 22 ± 3 g/m^2.7^, *p* <0.001) and the LV myocardium was 10 % thicker (5.6 ± 0.8 mm vs 5.1 ± 0.8 mm, *p* <0.001) in the obese/overweight children. This evidence of cardiac remodeling was present in obese children as young as age 8. Twenty four percent of obese/overweight children had concentric hypertrophy, 59 % had normal geometry and 17 % had either eccentric hypertrophy or concentric remodeling. LV mass index, thickness, ejection fraction and peak longitudinal and circumferential strains all correlated with epicardial adipose tissue after adjusting for height and gender (all *p* <0.05). Peak longitudinal and circumferential strains showed a significant relationship with the type of LV remodeling, and were most impaired in children with concentric hypertrophy (*p* <0.001 and *p* = 0.003, respectively).

**Conclusions:**

Obese children show evidence of significant cardiac remodeling and dysfunction, which begins as young as age 8. Obese children with concentric hypertrophy and impaired strain may represent a particularly high risk subgroup that demands further investigation.

**Electronic supplementary material:**

The online version of this article (doi:10.1186/s12968-016-0247-0) contains supplementary material, which is available to authorized users.

## Background

Obesity in the United States is an epidemic. One in three adults and nearly one in five children are considered obese according to their body mass index (BMI) [1, 2]. Unfortunately, many health problems associated with adult obesity are also observed in obese children, including changes in cardiac geometry and function. In adults, changes in cardiac geometry (e.g. hypertrophy) [3] and function [4] are associated with an increased risk of mortality.

The most consistently observed change in the hearts of obese children is greater myocardial mass (hypertrophy) [5]. In adults, hypertrophic remodeling is typically subcategorized using chamber dimensions and relative wall thickness (RWT), and different risk profiles have been observed among these categories: the concentric form of hypertrophy (increased mass and wall thickness) has a stronger association with mortality compared to eccentric hypertrophy (increased mass and normal wall thickness) and concentric remodeling (normal mass and increased wall thickness) [6].

Characterization of hypertrophy in the pediatric obese heart in this way has been included in few study designs. In one study, 42 % of obese children with normal blood pressure had concentric remodeling and 23 % had concentric hypertrophy, but obese children with hypertension presented with almost double the amount of concentric hypertrophy compared to their normotensive peers. No specific association between remodeling and contractile dysfunction (fractional shortening) was observed [7]. However, this study and others using M-mode are limited by the restricted data points used in RWT calculations and the high dependency on the angle of the echocardiographic imaging plane. Cardiovascular magnetic resonance (CMR) can accurately assess myocardial mass and thickness [8]. This study leverages the superior accuracy of CMR to better characterize left ventricular (LV) hypertrophy in obese children and to explore associations between remodeling and cardiac function.

**Fig. 1:**
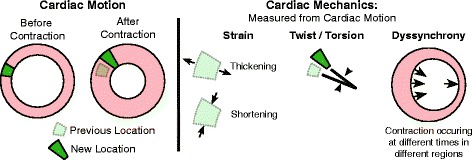
Cardiac mechanics. As the heart contracts, myocardial tissue displaces over time (cardiac motion). This motion or change in length of the myocardial tissue (strain) can be quantified using DENSE imaging. Additionally, the heart has a twisting motion which can be captured using DENSE. Dyssynchrony in LV contraction can also be measured using DENSE

The most widely used measure of cardiac function is ejection fraction (EF). Studies reporting EF in obese children are inconsistent with separate findings of increases [9], decreases [10], or no changes [11] using echocardiography. As an alternative to EF, cardiac mechanics (strain, torsion, and synchrony of contraction, Fig. 1) are more advanced and sensitive measures of function that are also better predictors of mortality [4]. There is evidence that cardiac mechanics may detect contractile dysfunction preceding clinical manifestations of disease, making mechanics ideal for monitoring asymptomatic obese children [12]. Recently, a few studies have reported impaired cardiac mechanics (strain) in obese children using echocardiography [13, 14].

It is important to note that no studies to our knowledge have examined the associations between remodeling types and advanced measures of function derived from cardiac mechanics in obese children. This understanding could be an important link in helping to risk stratify obese, pediatric patients in a clinical setting. Also, no study has reported a comprehensive analysis of the LV, including remodeling and mechanics, and their association with adiposity using CMR in obese children. Finally, CMR is the gold standard for quantifying body composition, particularly visceral and epicardial adipose tissue, which may have negative implications on cardiac function [15].

The goal of our study was to elucidate the changes in cardiac geometry and function in obese children ages 8–18 by using CMR and to determine whether the changes in specific remodeling types are associated with changes in cardiac function. In addition, we determined inter-observer and inter-test reproducibility of the measures of cardiac geometry and mechanics derived from CMR in obese children.

## Methods

### Study population

Obese (BMI ≥95^th^ percentile), overweight (BMI 85^th^–95^th^ percentile) and healthy weight (BMI 5^th^–85^th^ percentile) children [16] ages 8–18 years were prospectively enrolled. Exclusion criteria included: 1) diabetes, diagnosed hypertension, history of heart disease; 2) contraindications for CMR; 3) waist circumference >125 cm due to the circumference limitation of the CMR bore.

### Ethics, consent and permissions

The Institutional Review Boards at both the University of Kentucky (13-0201-P6H) and Geisinger Health System (2015-0159) approved the study, and all subjects provided assent and had written and informed consent by their parents/legal guardians.

### Clinical assessment

At the time of the CMR, height and weight, averaged from two readings, were measured and BMI (weight/height^2^ in kg/m^2^) percentiles based on CDC growth charts [17] were determined. Resting blood pressure (average of two readings taken 5 min apart while seated for at least 10 min) by auscultation using an appropriately sized cuff was also performed. All children had a normal 12-lead electrocardiogram.

To assess the accuracy of our clinical measure of blood pressure, a subset of 23 subjects underwent ambulatory blood pressure monitoring every 30 min over a 24-hour period using an appropriately sized cuff according to standard guidelines [18]. Overall blood pressure by averaging all readings over the 24 h period was reported and compared to the clinical measure of blood pressure.

### CMR

A detailed description of image acquisition and analysis is provided in the Additional file 1.

#### Ventricular mass, volumes, and ejection fraction

**Fig. 2:**
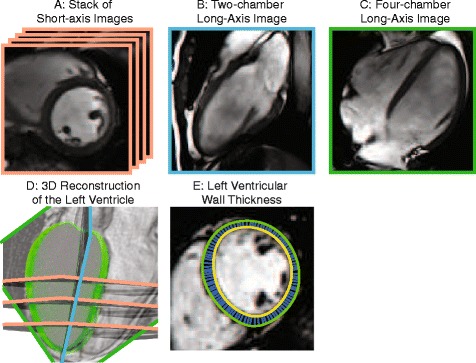
Quantifying myocardial mass, volumes, and thickness in 3D. A short axis stack of SSFP images (**a**), a two-chamber view (**b**), and a four-chamber view (**c**) were acquired. Contours were drawn around the LV endocardial and epicardial borders and a 3D model of the LV (**d**) was computed. Additionally, 3D LV thickness was calculated from over 2000 data points (**e**)

All subjects underwent CMR on a 3T Siemens Tim Trio (Erlangen, Germany) using 6-element chest and 24-element spine coils. Imaging parameters are provided in the Additional file 1: Table S1. Standard two and four-chamber steady-state free-precession (SSFP) images and a short-axis stack of cine SSFP images spanning the LV were acquired (Fig. 2a-c). LV end-diastolic and end-systolic volumes, mass, EF (Fig. 2d), and myocardial thickness via 3D modeling (Fig. 2e) were calculated using custom software written in MATLAB (Mathworks, Inc., Natick, MA). LV mass was indexed to height (LVMI, grams/meters^2.7^) [19]. RWT was also assessed along a 1-dimensional line projected through the 3D model to facilitate comparison of our 3D measures of myocardial thickness to published data from echocardiography:

$$ \mathrm{R}\mathrm{W}\mathrm{T}=\frac{\mathrm{posterior}\ \mathrm{wall}\ \mathrm{thickness}+\mathrm{septal}\ \mathrm{wall}\ \mathrm{thickness}}{\mathrm{LV}\ \mathrm{end}\ \mathrm{diastolic}\ \mathrm{dimension}} $$

Cardiac remodeling types were established based on the healthy cohort’s 95^th^ percentile values for mass/volume ratio and LVMI. A bootstrap technique was used to define the 95 % confidence interval of the percentile cutoffs. Children were classified into four categories of remodeling according to the following rules:

1. Normal = below the 95^th^ percentile for both LVMI and mass/volume ratio
2. Concentric remodeling = above the 95^th^ percentile for mass/volume ratio only
3. Eccentric hypertrophy = above the 95^th^ percentile for LVMI only
4. Concentric hypertrophy = above the 95^th^ percentile for both LVMI and mass/volume ratio

#### Cardiac mechanics

Spiral cine Displacement Encoding with Stimulated Echoes (DENSE) imaging was used to quantify cardiac deformation [20]. Basal, mid-ventricular, and apical short-axis DENSE images planned at end-systole and two- and four-chamber long-axis views planned at end-diastole were acquired with a respiratory navigator. Strains, torsion, and dyssynchrony were calculated from DENSE phase images using custom MATLAB software. LV dyssynchrony was quantified using the circumferential and radial uniformity ratio estimates (CURE and RURE) [21].

#### Diastolic function

Blood flow velocity through the mitral valve was measured using phase contrast CMR during free breathing. Early (E) and late (A) diastolic filling velocities were quantified from the phase contrast images, and the velocity of the mitral annulus (E’) was quantified from the four-chamber DENSE images.

#### Adipose tissue

**Fig. 3:**
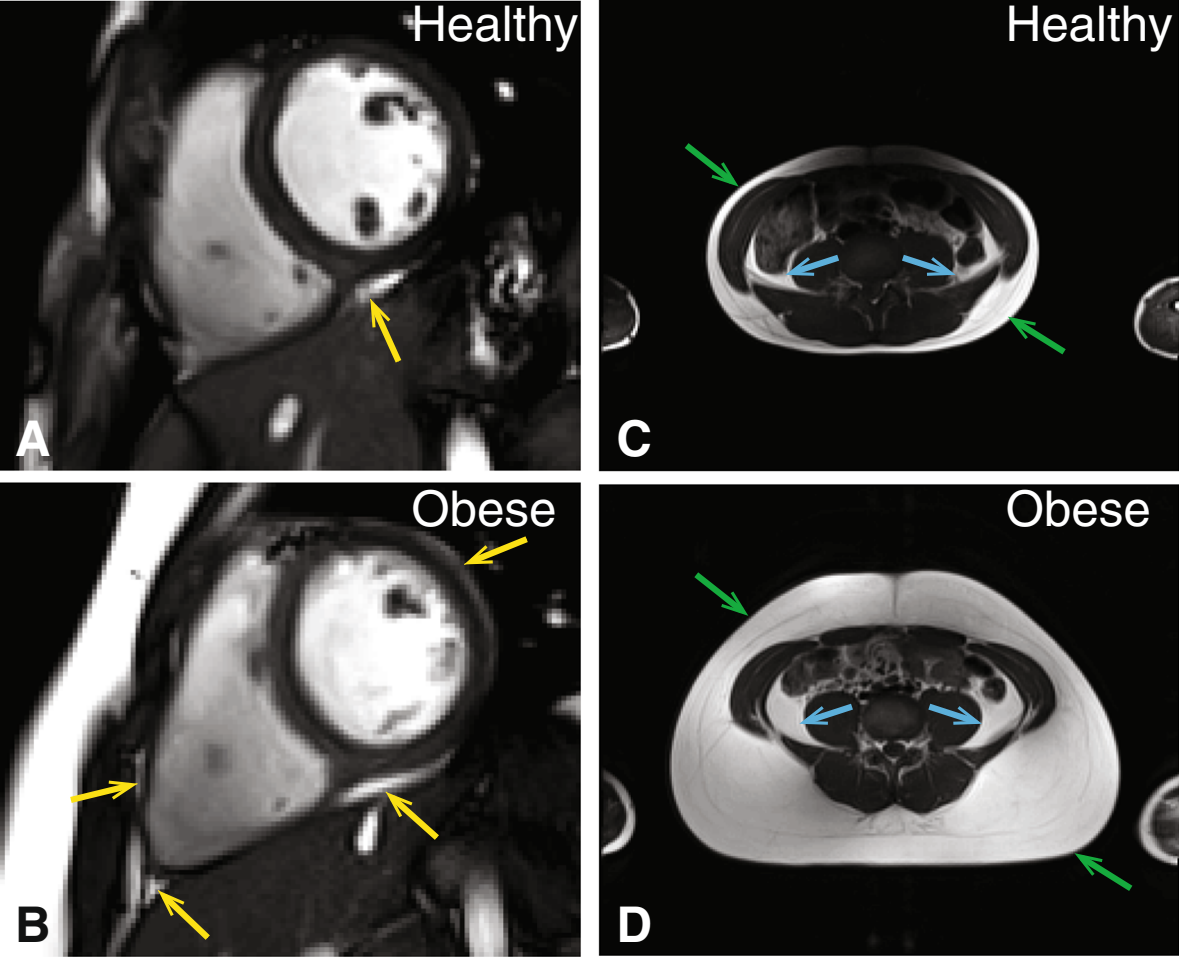
Obese children have more epicardial and abdominal adiposity. The top row shows images from a healthy weight (70^th^ percentile) 12 year-old male and the bottom row shows images from an obese (98^th^ percentile) 12 year-old male. Panels **a** and **b** show epicardial adipose tissue on a representative short-axis view of the heart. Panels **c** and **d** have arrows indicating the subcutaneous and visceral layers of adipose tissue from a transverse view of the L4/L5 disc

The 3D volume of epicardial adipose tissue (EAT) was quantified using the short-axis stack of cine SSFP images (Fig. 3a/b) spanning both ventricles. A transverse T1-weighted image at the L4-L5 vertebrae was acquired at end-expiration to quantify areas of visceral (blue arrows) and subcutaneous (green arrows) adipose tissue (VAT and SAT, Fig. 3c/d).

### Reproducibility

#### Inter-observer

CMR derived cardiac geometry and mechanics data from ten randomly selected obese subjects were analyzed by two different investigators.

#### Inter-test

Nine obese subjects were scanned twice to quantify inter-test reproducibility. Four subjects were scanned twice on the same day, three were scanned twice within the same week, and two had a repeat scan within 6 weeks. For the second scan, subjects were removed from the scanner, new electrocardiogram leads were placed, and a different technician collected a complete set of new images using the same protocol.

### Statistics

Modified coefficient of variation (CoV) was used to quantify reproducibility [22]. Continuous variables from obese/overweight and healthy groups were compared with a student’s t-test and presented as mean ± standard deviation (SD). Sex differences were determined using the Fisher’s exact test. Linear regression was used to estimate the differences in cardiac remodeling and function between groups, while accounting for the effect of age. Bonferroni’s correction was used to control for multiple comparison. The interaction between group and age was tested and, if found to be significant, it was retained in the model.

Multivariable linear regression was used to estimate the association between adiposity (estimated by BMI z-score, EAT and VAT) and specific cardiac outcomes (cardiac geometry, function and strains). Height and sex were included in the model to account for somatic growth and sex difference. To determine whether obesity had an association with cardiac remodeling (LVMI and thickness) independent of blood pressure, these outcomes were also adjusted for blood pressure in addition to height and sex.

Analysis of Variance (ANOVA) was used to test for differences between cardiac strains among the different remodeling types. Dunnett’s multiple comparison procedure was used to control for multiple testing. The concentric remodeling and eccentric hypertrophy groups were combined to improve power. The normal geometry group was compared to the concentric hypertrophy group and to the combined concentric remodeling/eccentric hypertrophy group. Statistical significance was defined as *p* ≤0.05.

## Results

### Study population, clinical assessment, and body composition

This study included 41 obese/overweight children (32 obese, 9 overweight; age 12 ± 3 years, 56 % female) and 29 healthy children (age 14 ± 3 years, 41 % female) with characteristics shown in Table 1. Systolic and mean arterial pressures were mildly elevated by 7 and 5 % (*p* = 0.006 and 0.04), respectively, in the obese/overweight group. In addition, obese/overweight children had more than double the subcutaneous, visceral and epicardial adipose tissue compared to healthy controls (*p* <0.001).

**Table 1:** Clinical and demographic parameters, and body composition of the study population

	Obese/Overweight *n* = 41	Healthy *n* = 29	*p* value
*Age (years)*	12 ± 3	14 ± 3	0.04
*Sex (M/F)*	18/23	17/12	0.33
*Weight (kg)*	73 ± 23	52 ± 15	<0.001
*Height (cm)*	157 ± 11	164 ± 18	0.05
*Body Mass Index (kg/m* ^*2*^ *)*	29 ± 6	19 ± 2	<0.001
*Body Mass Index Percentile*	97 ± 3	42 ± 24	<0.001
*Body Mass Index z-score*	2.0 ± 0.4	−0.3 ± 0.8	<0.001
*Heart rate (beats/min)*	74 ± 11	70 ± 8	0.09
*Systolic blood pressure (mmHg)*	116 ± 13	108 ± 8	0.006
*Diastolic blood pressure (mmHg)*	73 ± 7	71 ± 6	0.25
*Mean arterial pressure (mmHg)*	87 ± 8	83 ± 7	0.04
*Subcutaneous adipose tissue (cm* ^*2*^ *)*	385 ± 172	89 ± 48	<0.001
*Visceral adipose tissue (cm* ^*2*^ *)*	53 ± 23	17 ± 10	<0.001
*Epicardial adipose tissue (cm3)* ^a^	32 ± 27	14 ± 9^b^	<0.001

^a^Epicardial adipose tissue is in units of cm^3^ since it was quantified using a 3D stack of SSFP images. All other measures were derived from a single imaging plane and are therefore reported as areas (cm^2^)

^b^One of the 29 healthy weight subjects did not complete cine imaging and was therefore not included in the comparison of epicardial adipose tissue

### Ventricular mass, volumes, cardiac function and mechanics

**Fig. 4:**
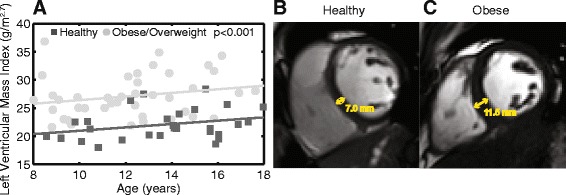
Obese children have greater left ventricular mass index (LVMI) and myocardial thickness. The greater LVMI in obese children is apparent across all ages (**a**). Representative examples are shown in 14 year-old healthy (43^rd^ percentile, **b**) and obese (99^th^ percentile, **c**) females

After adjusting for age, obese/overweight children had a 23 % greater LVMI compared to healthy controls (27 ± 4 g/m^2.7^ vs 22 ± 3 g/m^2.7^, *p* <0.001, Table 2). This difference in mass was evident in obese/overweight children as young as 8 years old (Fig. 4a). In addition to a greater mass, the average LV thickness was 10 % higher in the obese/overweight group compared to healthy controls (5.6 ± 0.8 mm vs 5.1 ± 0.8 mm, *p* <0.001, age adjusted). A representative difference in myocardial thickness is shown in Fig. 4b and c. Additionally, obese/overweight children had a larger LV mass/volume ratio and RWT compared to controls. There were no differences in EF, LV volumes or diastolic function between the two groups (Table 2).

**Table 2:** Cardiac geometry and function

	Obese/Overweight *n* = 41	Healthy *n* = 29^b^	*p* value	*p* value, age adjusted
Cardiac geometry				
*LV mass (g)*	92 ± 23	87 ± 29	0.45	0.001
*LV mass index (g/m* ^*2.7*^ *)*	27 ± 4	22 ± 3	<0.001	<0.001
*LV end systolic volume (mL)*	51 ± 14	55 ± 19	0.47	0.36
*LV end diastolic volume (mL)*	135 ± 30	141 ± 41	0.47	0.30
*LV mass/volume ratio (dimensionless)*	0.68 ± 0.09	0.61 ± 0.05	<0.001	<0.001
*Relative wall thickness (dimensionless)*	0.28 ± 0.04	0.26 ± 0.04	0.05	0.006
*Average LV thickness (mm)*	5.6 ± 0.8	5.1 ± 0.8	0.01	<0.001
*Maximum LV thickness (mm)*	8.9 ± 1.3	8.0 ± 1.2	0.004	<0.001
*90* ^*th*^ *percentile of LV thickness (mm)* ^a^	7.4 ± 1.0	6.8 ± 1.0	0.01	<0.001
Cardiac function				
*LV ejection fraction (%)*	62 ± 4	62 ± 4	0.98	0.51
*E wave velocity (cm/s)*	61 ± 11	65 ± 13	0.17	0.16
*A wave velocity (cm/s)*	28 ± 12	30 ± 13	0.51	0.52
*E/A ratio (dimensionless)*	2.5 ± 1.0	2.5 ± 1.0	0.97	0.88
*E’ (cm/s)*	12 ± 4	11 ± 2	0.78	0.93
*E/E’ ratio (dimensionless)*	5.9 ± 2.9	5.9 ± 1.8	0.92	0.80

*LV* left ventricular.

^a^This was derived by measuring LV thickness in 3D space at thousands of points throughout the ventricle for each subject, and taking the 90^th^ percentile of all these measurements

^b^One of the 29 healthy weight subjects did not complete cine imaging and was therefore not included in any of the measures of cardiac geometry or LV ejection fraction

After accounting for age, an impairment in peak longitudinal strain was observed in obese/overweight children compared to their healthy weight peers (−14 ± 2 % vs −15 ± 2 %, *p* = 0.02), while no differences were seen in peak circumferential or radial strain. Obese/overweight children also had a lower circumferential diastolic strain rate (1.00 ± 0.22 s^−1^ vs 1.11 ± 0.27 s^−1^, *p* = 0.03) compared to the controls. There were no differences in other strain rates, torsion, or measures of synchrony (Table 3).

**Table 3:** Cardiac mechanics

	Obese/Overweight *n* = 39^a^	Healthy *n* = 29	*p* value	*p* value, age adjusted
Peak strain				
*Circumferential (%)*	−19 ± 2	−19 ± 2	0.77	0.37
*Radial (%)*	31 ± 12	33 ± 10	0.68	0.41
*Longitudinal (%)*	−14 ± 2	−15 ± 2	0.09	0.02
Peak systolic strain rate				
*Circumferential (s* ^*−1*^ *)*	−1.17 ± 0.99	−1.05 ± 0.69	0.56	0.36
*Radial (s* ^*−1*^ *)*	1.86 ± 1.29	1.74 ± 1.00	0.67	0.36
*Longitudinal (s* ^*−1*^ *)*	−0.77 ± 0.52	−0.94 ± 0.59	0.21	0.39
Peak diastolic strain rate				
*Circumferential (s* ^*−1*^ *)*	1.00 ± 0.22	1.11 ± 0.27	0.09	0.03
*Radial (s* ^*−1*^ *)*	−1.95 ± 0.81	−1.91 ± 1.03	0.84	0.59
*Longitudinal (s* ^*−1*^ *)*	0.90 ± 0.41	0.87 ± 0.27	0.76	0.66
Cardiac Torsion/Synchrony				
*Torsion (degrees/cm)*	4.0 ± 0.8	3.6 ± 1.0	0.11	0.56
*CURE (dimensionless)*	0.97 ± 0.03	0.97 ± 0.02	0.50	0.86
*RURE (dimensionless)*	0.83 ± 0.10	0.81 ± 0.12	0.54	0.26

^a^Two of the 41 obese subjects did not complete DENSE imaging and therefore did not have cardiac mechanics available for comparison

### Association between cardiac remodeling and strain

**Fig. 5:**
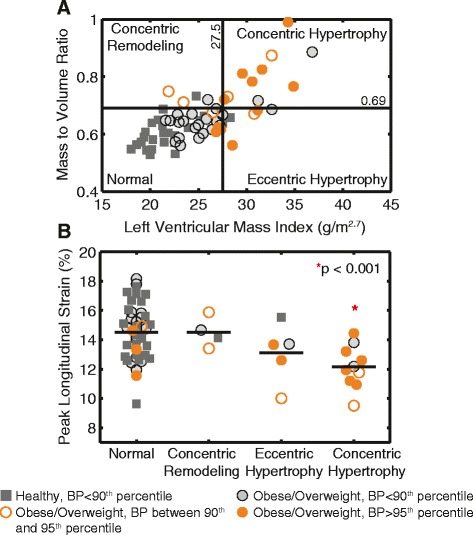
Cardiac remodeling is associated with longitudinal strain. **a** classification of cardiac remodeling based on left ventricular mass index (LVMI) and mass/volume ratio, colored by blood pressure (BP). Cutoff values of LVMI (27.5) and mass/volume ratio (0.69) are based on the 95^th^ percentile of the healthy weight controls. **b** children with concentric hypertrophy had impaired longitudinal strain

Figure 5a highlights the types of cardiac remodeling observed in the study population. The 95^th^ percentile cutoff for mass/volume ratio derived in the healthy weight cohort was 0.69 (95 % confidence interval = 0.64 to 0.73) and the 95^th^ percentile cutoff for LVMI was 27.5 g/m^2.7^ (95 % confidence interval = 25.9 to 29.1). In the obese/overweight group, ten children (24 %) had concentric hypertrophy, seven (17 %) had either concentric remodeling or eccentric hypertrophy, and the remaining 24 children (59 %) had normal geometry. Table 4 summarizes the peak strain values in each remodeling category, and Fig. 5b shows peak strain values for each of the subjects in their respective remodeling category. Compared to children with normal geometry, those with concentric hypertrophy showed impairments in both peak longitudinal (−15 ± 2 % vs −12 ± 1 %, *p* <0.001) and circumferential (−19 ± 2 % vs −17 ± 2 %, *p* = 0.003) strain. There was no difference in strains between the normal geometry group and the combined concentric remodeling/eccentric hypertrophy group.

**Table 4:** Peak strain values for different remodeling types

Peak strain	Normal^c^ *n* = 48	Concentric Remodeling/Eccentric hypertrophy *n* = 9	*p* value^a^	Concentric hypertrophy *n* = 10	*p* value^b^
Circumferential	−19 ± 2	−18 ± 2	0.51	−17 ± 2	0.003
Radial	31 ± 10	31 ± 14	1.00	35 ± 9	0.55
Longitudinal	−15 ± 2	−14 ± 2	0.39	−12 ± 1	<0.001

^a^Concentric Remodeling/Eccentric Hypertrophy vs Normal

^b^Concentric Hypertrophy vs Normal

^c^Two obese subjects in the normal remodeling group did not complete DENSE imaging and therefore did not have strains available for comparison and one subject in the healthy weight group did not complete cine imaging so is not included in the remodeling type analysis

**Fig. 6:**
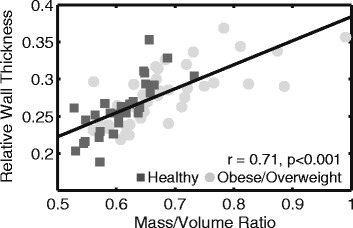
Relative wall thickness and mass/volume ratio. Relative wall thickness derived from CMR correlates with 3D mass/volume ratios

RWT was positively correlated with mass/volume ratio (*r* = 0.71, *p* <0.001, Fig. 6). Using the 95^th^ percentile of the healthy subjects as a cutoff value for mass/volume (x = 0.69), we calculated the appropriate upper limit for RWT in obese children using the linear equation from Fig. 6 to be 0.28.

### Associations between adiposity and cardiac geometry and function

Table 5 summarizes the associations between adiposity (estimated by BMI z-score, EAT and VAT) and cardiac geometry, function and strains. Sex and height were controlled in the multivariate model to account for somatic growth.

**Table 5:** Associations (Correlation Coefficients) between Cardiac Outcomes and Adiposity

	BMI z-score		EAT		VAT	
	*Unadjusted*	*Adjusted* ^*a*^	*Unadjusted*	*Adjusted* ^*b*^	*Unadjusted*	*Adjusted*
Geometry						
LVMI	0.69**	0.71**	0.39**	0.43**	0.50**	0.54**
Average thickness	0.43**	0.46**	0.47**	0.48**	0.42**	0.57**
Function						
EF	0.003	−0.008	−0.27*	−0.24*	−0.09	−0.11
E/A	−0.04	−0.01	−0.03	−0.01	−0.11	−0.07
E/E’	−0.02	−0.02	−0.05	−0.08	−0.07	−0.08
Peak strain						
Circumferential	0.15	0.18	0.29*	0.29*	0.22	0.28*
Radial	−0.03	−0.06	−0.03	−0.02	−0.10	−0.14
Longitudinal	0.23	0.22	0.40**	0.36*	0.31*	0.30

*LVMI* left ventricular mass index, *BMI* body mass index, *EAT* epicardial adipose tissue, *VAT* visceral adipose tissue, *EF* ejection fraction. *p <0.05; **p <0.001. ^a^BMI z-score is adjusted for sex; ^b^EAT and VAT are adjusted for height and sex

LVMI and myocardial thickness were correlated with all three measures of adiposity. Ejection fraction was correlated with EAT but not BMI z-score or VAT. No significant correlations were seen between measures of diastolic function and adiposity.

**Fig. 7:**
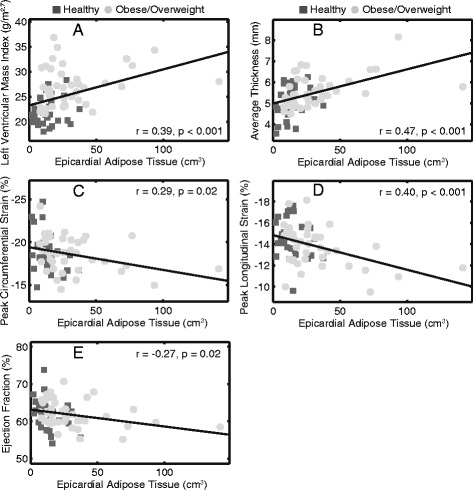
Measures of cardiac geometry and function correlate with epicardial adipose tissue. **a** left ventricular mass index (**b**) average thickness (**c**) peak circumferential strain (**d**) peak longitudinal strain (**e**) ejection fraction

Peak circumferential and longitudinal strains were correlated with both EAT and VAT, but not BMI z-score. Figure 7 shows examples of correlations between cardiac outcomes and EAT.

### Blood pressure and cardiac remodeling

**Fig. 8:**
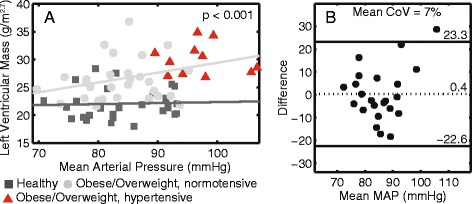
Left ventricular mass index is associated with obesity independent of blood pressure. **a** Correlation between left ventricular mass index and blood pressure. Children with hypertension were highlighted (*red triangles*). **b** Mean arterial pressure (MAP) measured at clinical assessment agrees with 24-hour ambulatory blood pressure monitoring

After adjusting for mean arterial pressure, height and sex, LVMI and average wall thickness remained significantly correlated with BMI z-score, EAT and VAT (Table 6). Figure 8a illustrates the relationship between LVMI and mean arterial pressure. LVMI of the obese/overweight children plotted on a different line from the healthy weight controls, which illustrates the association between LVMI and obesity independent of blood pressure.

**Table 6:** Associations between Cardiac Geometry and Adiposity after Accounting for Blood Pressure

Cardiac outcome	BMI z-score (Sex and MAP Adjusted)		EAT (Sex, Height and MAP Adjusted)		VAT (Sex, Height and MAP Adjusted)	
	r	*p*	r	*p*	r	*p*
LVMI	0.69	<0.001	0.32	0.01	0.40	0.001
Average Thickness	0.39	0.001	0.36	0.003	0.42	<0.001

*LVMI* left ventricular mass index, *MAP* mean arterial pressure

Figure 8b shows that mean arterial pressure measured clinically strongly agrees with that measured by 24-hour ambulatory monitoring (85 ± 8 mmHg vs 86 ± 11 mmHg, CoV = 7 %). Similar results were also observed for systolic and diastolic blood pressure (both CoVs = 10 %).

### Reproducibility

Inter-observer reproducibility was excellent with the exception of longitudinal strain rates. Inter-test reproducibility was also excellent for all parameters of cardiac geometry and particularly all peak strain measurements (Table 7). All CoVs, limits of agreement, and biases are provided in Additional file 1: Table S2.

**Table 7:** Inter-test reproducibility

	CoV%	+/−2SD Limits	Bias
Cardiac geometry and function			
*LV mass (g)*	5	−19, 9	−5.0
*LV end systolic volume (mL)*	6	−11, 8	−1.5
*LV end diastolic volume (mL)*	3	−17, 15	−0.8
*Average thickness (mm)*	5	−1, 0.4	−0.3
*Ejection Fraction (%)*	4	−7, 9	1.0
Peak strain			
*Circumferential (%)*	5	−3.2, 2.7	−0.2
*Radial (%)*	12	−10, 19	−4.5
*Longitudinal (%)*	12	−5, 5	−0.2

*LV* left ventricular, *CoV* coefficient of variation

## Discussion

We prospectively enrolled 41 obese/overweight children (BMI ≥85^th^ percentile) and 29 healthy weight controls (BMI 5^th^–85^th^ percentile) to understand and characterize changes in cardiac geometry and function in the setting of pediatric obesity. Our major findings were: 1) children with obesity as young as 8 years old have larger LVMI and average myocardial thickness; 2) 24 % of obese/overweight children have concentric hypertrophy, and these children with concentric hypertrophy have the lowest peak longitudinal and circumferential strains; 3) measures of cardiac geometry, function and mechanics correlate with measures of adiposity; 4) the elevation in LVMI and myocardial thickness in obese/overweight children was only partially explained by and is independent of blood pressure; and 5) inter-test reproducibility for assessing cardiac geometry and mechanics using CMR in obese children is overall very good.

### Cardiac function and mechanics

Most studies agree that obese children have a larger LVMI. Only a handful of studies have measured cardiac strain (all using Doppler derived strains), and the results are inconsistent. Measuring cardiac strain with echocardiography can be difficult due to the angle-dependency of Doppler Imaging and the physical interference of excess adiposity reducing image quality [23]. Previous studies have reported decreased longitudinal strain [13, 14], decreased [14] or no change [13] in circumferential strain, and increased [14], decreased [13], or no change [5] in radial strain. Most of these studies only report the strain values from the most visible areas of the heart (posterior wall and septum). With CMR, we were able to reproducibly quantify cardiac mechanics in the longitudinal, circumferential, and radial directions throughout the entire left ventricle without the errors introduced by angle-dependency and excess adiposity [22].

Our results showed an impairment in peak longitudinal strain in obese/overweight children after adjusting for age, which is in agreement with other studies [13, 14]. Furthermore, we found that obese children with concentric hypertrophy had significantly impaired circumferential and longitudinal strain. Using a highly accurate technique, such as DENSE CMR, bolsters the evidence that obese children develop cardiac contractile dysfunction early in life, as young as 8 years of age.

Limited information on torsion or measures of synchrony in obese children has been reported. Saltijeral et al. found an increase in torsion among obese children compared to healthy controls [14], and implied that this occurs in order to maintain cardiac output in the presence of decreased contractile function. Alternatively, these measures were shown to be reduced in animal models of diet-induced obesity [24]. However, we observed no changes in torsion or synchrony in obese children compared to healthy weight controls. Longitudinal studies are necessary to determine whether these measures of cardiac mechanics are impaired in the setting of prolonged obesity.

Discrepant results have been reported on cardiac contractile function quantified by ejection fraction. In the current study, we did not see an overall difference in ejection fraction between the obese/overweight and healthy weight groups. However, we observed a weak correlation between ejection fraction (*r* = 0.24, *p* = 0.02, Table 5) and EAT. The inconsistent results in previous studies could be attributed to variations in sample size and study population.

An impairment in diastolic function in obese/overweight children was evidenced by a lower circumferential diastolic strain rate (Table 3). However, no differences were observed in longitudinal or radial diastolic strain rate. Also, we did not observe any changes in common echocardiographic measures of diastolic function, such as E/A and E/E’ ratio. This may be due to the relatively low temporal resolution of CMR, which may be particularly important when quantifying diastolic function. Our effective temporal resolution was 17 ms with view sharing in the current study.

### Cardiac remodeling

The sensitivity of CMR to delineate cardiac boundaries facilitates highly accurate quantification of cardiac mass, volumes, and thickness. There were no group differences in chamber size at end diastole or end systole, but LVMI was significantly greater in the obese group. We investigated the type of cardiac remodeling present by comparing the mass/volume ratio across subjects. Twenty-four percent of the obese/overweight children had concentric hypertrophy (Fig. 5), which is the remodeling type most closely related to mortality [6]. It is worth noting that although 11 of the obese/overweight children had a clinical blood pressure measurement above the 90^th^ percentile, some obese/overweight children with blood pressure over the 90^th^ percentile also had normal geometry, eccentric hypertrophy or concentric remodeling (Fig. 5). Along with the fact that LVMI followed two separate trend lines between the obese/overweight and healthy weight groups when plotted against blood pressure (Fig. 8), this suggests that blood pressure is not the only determinant of the larger LVMI among obese/overweight children.

We also found that children with concentric hypertrophy had contractile dysfunction as evidenced by impaired circumferential and longitudinal strain. The combined hypertrophy and impaired contractile function in the heart may increase the risk of cardiovascular disease and mortality among children with obesity. It may be clinically relevant to assess cardiac remodeling types in obese children as part of their cardiovascular risk profile. This group of children with impaired strain and concentric remodeling may have the highest risk of adverse outcomes and potentially need to be targeted differently in clinic.

As expected, RWT was not perfectly correlated with mass/volume values from CMR (Fig. 6). This is likely because RWT assesses a 3D structure using 1-dimensional data. Using RWT to characterize the types of remodeling in obese children may therefore be imprecise. More comprehensive measures from 3D CMR reconstructions like 3D mass/volume may be essential to accurately quantify cardiac remodeling and therefore help to risk stratify children with obesity for treatment in a clinical setting. Additionally, using the values derived from 3D mass/volume, we calculated the upper cutoff for RWT in obese children to be 0.28. This limit has been reported previously using echocardiography as 0.375 [25]. With the superior accuracy of CMR in delineating cardiac borders compared to echocardiography, a RWT of 0.28 derived from CMR may be a more appropriate upper limit.

### Associations of adiposity with cardiac remodeling and dysfunction

Multivariate models accounting for height and sex were implemented to examine contributions of adiposity to cardiac remodeling and dysfunction independent of somatic growth. Results show that EAT was correlated to most measures of cardiac remodeling, function and mechanics, while BMI z-score and VAT were mostly associated with cardiac remodeling (LVMI and thickness) but not functional measures. This suggests that excessive EAT may be a stronger indicator for cardiac remodeling and dysfunction compared to BMI z-score and VAT.

### Reproducibility

Overall, inter-observer reproducibility was good with the exception of peak longitudinal strain rates (Additional file 1: Table S2). Inter-test reproducibility was excellent for all measures of cardiac geometry and for measures of peak strains in particular, but was not as good for radial and longitudinal strain rates. Poor reproducibility of strain rate has been previously reported [22] and could be a fundamental limitation of calculating strain rates from myocardial displacement (which requires two derivatives). However, speckle-tracking echocardiography or any technique that derives strain rates from measured displacement would suffer from the same limitation. To our knowledge, this is the first study to assess and show good inter-*test* reproducibility of cardiac mechanics in obese children. It is highly important to establish reproducibility of these methods prior to using a technique to assess children before and after an intervention. We therefore believe the superior reproducibility of CMR will make it an increasingly favorable modality for monitoring obese children.

### Limitations

This was a cross-sectional study. Longitudinal studies need to delineate the timeline of the development of heart disease in obese children, as well as the potential for reversing these changes. In addition, younger children should be monitored for these changes, as obese children younger than 8 years old likely also have signs of heart disease.

We did not collect any blood to measure lipids, inflammation, or insulin resistance. It is possible that obese children have increased insulin signaling and that insulin-mediated activation of Akt in the heart contributes to hypertrophy [26]. Future studies should measure insulin resistance to elucidate the impact that insulin and its downstream targets may play in cardiac remodeling in obese children.

Blood pressure was measured using a single clinical assessment. This may be problematic for several reasons. First, only two readings were taken instead of taking three measurements and discarding the first measure. However, caution was taken to ensure there was only small differences between the two readings, otherwise a third reading was taken and the first measure was discarded. Second, brachial blood pressure in children is highly variable, therefore, a single assessment may misrepresent and underestimate the effect of blood pressure on cardiac remodeling. However, we performed 24-hour ambulatory blood pressure monitoring in a subset of 23 children, and their blood pressures by the two methods showed good agreement. Third, children with diagnosed hypertension were excluded from the study due to the difficulty of controlling for the effects of treatment. This may truncate the effect of blood pressure on outcomes, as well as affect the proportion of obese children with different types of remodeling.

Severely obese subjects could not be enrolled in this study due to size constraints of our CMR scanner. A more severely obese population may show even more cardiac remodeling or dysfunction than what we have already documented. Future studies should include children with all levels of obesity, accommodated by a wide bore CMR scanner.

We defined the cutoff values for LVMI and mass/volume ratio using the 29 healthy weight subjects in our study since there are no established CMR values for children. The small sample size may result in an underestimation of the upper limits for the entire population. Future studies with a larger cohort may establish more accurate cutoff values for children using CMR.

## Conclusion

Children with obesity have greater left ventricular mass index and impaired contractile function, both related to excessive adiposity, even as young as 8 years old. There is an association between the type of cardiac remodeling and cardiac function, with children who have concentric hypertrophy showing the most impaired circumferential and longitudinal strain. The detailed analysis of the heart from CMR sheds light on discrepancies observed in previous studies about childhood obesity and heart disease. These findings provide support for the use of CMR in a clinical and research setting to evaluate cardiovascular risk in obese children.

## Additional file

Additional file 1: Supplemental Material. (PDF 72 kb)

## Abbreviations

BMI: body mass index
CMR: cardiovascular magnetic resonance
LV: left ventricular
EF: ejection fraction
RWT: relative wall thickness
SSFP: steady-state free-precession
DENSE: displacement encoding with stimulated echoes
LVMI: left ventricular mass index
EAT: epicardial adipose tissue
VAT: visceral adipose tissue
SAT: subcutaneous adipose tissue
CoV: coefficient of variation
SD: standard deviation
MAP: mean arterial pressure
