# Supplementary material for: Cardiac remodeling and dysfunction in childhood obesity: a cardiovascular magnetic resonance study
Source: J Cardiovasc Magn Reson. 2016 May 11;18:28. doi: 10.1186/s12968-016-0247-0 (PMC4863365; doi:10.1186/s12968-016-0247-0)
Supplement: Additional file 1: — Supplemental Material. (PDF 72 kb) [file 12968_2016_247_MOESM1_ESM.pdf]

Supplemental Material

Study Population

Subjects were recruited from the High BMI Diagnostic Clinic at the University of Kentucky, the Center for Clinical and Translational Science volunteer databases and Geisinger Medical Center. Healthy controls were age- and sex- matched to the obese children in the study.

Cardiac MR Imaging

Ventricular Mass, Volumes, and Ejection Fraction

Depending on the size of the heart, a stack of 7–11 short-axis images was planned to cover both left and right ventricles. Slice thickness was 8 mm and slice gaps varied between 0 and 3.7 mm. Scans were ECG-gated in a retrospective fashion and acquired during an end-expiratory breath-hold. Reconstructed temporal resolution was 16.4–49.9 ms, and true temporal resolution was 33.9–50.6 ms.

Epicardial and endocardial boundaries of the LV were manually drawn on all images. These contours were then used to reconstruct 3D endocardial and epicardial surfaces.[1] These 3D models were used to directly compute LV volumes, LV myocardial mass (assuming a myocardial density of 1.05 gram/mL), and ejection fraction. The 3D reconstructions of the LV endo- and epicardium were used to measure myocardial thickness at over 2000 points throughout the ventricle using a PDE-based approach.[2] The posterior and septal wall thicknesses were measured on the LV outflow tract view generated from the 3D reconstruction of the heart, 1 cm below the valve plane, using a line plotted in 3D space similar to the methodology of echocardiography.

Cardiac Mechanics

Sequence parameters relevant to DENSE acquisition were: 6 or 18 spiral interleaves and in-plane simple displacement encoding (ke = 0.1 cycles/mm) with view-sharing, temporal resolution was 34 ms upsampled to 17ms with view sharing. The respiratory navigator acceptance window was set to +/- 3mm for a total range of 7mm – by default the Siemens software uses a 1mm window in the center of the acceptance window, which creates the total range of 7mm.

Analysis included manual segmentation of the myocardium, phase unwrapping, tissue tracking to derive the displacement of myocardial tissue throughout the cardiac cycle, and calculation of myocardial strains as previously described.[3–5] Regional strains were computed over the cardiac cycle and used to quantify LV synchrony using circumferential and radial uniformity ratio estimate (CURE and RURE) indices.[6]

Average strain curves from the base, mid and apical image slices were averaged together, and the systolic peak of this average curve was defined as the peak global strain in that particular direction. Peak longitudinal strain was averaged from the two and four-chamber views of the left ventricle. Average twist angles were computed for each of the three short-axis DENSE images and torsion was defined as the slope of the relationship between twist angle and longitudinal position (plotted for three points since we acquired three short-axis images).

Diastolic Function

Phase contrast images were acquired during free breathing using an image prescribed just below the mitral valve annulus visible in the four chamber SSFP image at end-systole. Velocities were encoded in the through-plane direction with a velocity encoding of 250 cm/s. Using a retrospective ECG-gated acquisition, 128 images were reconstructed over the cardiac cycle (reconstructed temporal resolution = 4.5–9.4 ms, true temporal resolution = 36.9 ms).

A circular region of interest (ROI) was drawn within the outflow region of the mitral valve and average velocity within this ROI was computed throughout the cardiac cycle. The E/A ratio was computed using the early diastolic (E) and late diastolic (A) peaks in the trans-mitral blood flow velocity. The velocity of the mitral annulus (E') was estimated using the displacement of the mitral annulus (averaged between the lateral and septal walls in basal segments 1 and 7 based on the standard segmentation of the long-axis LV) over the cardiac cycle measured from the four-chamber DENSE images.

Adipose Tissue Quantification and Classification

On the T1-weighted image, an ROI was drawn around the SAT, and the pixel area was measured in cm^2. An ROI was drawn around the VAT and a threshold was applied to measure pixel area. For EAT quantification, an ROI was drawn on each end-diastolic short-axis SSFP image. The 3D EAT volume (in cm^3) was measured by multiplying the pixel area with slice thickness after accounting for slice gaps.

References

1. Haggerty CM, Kramer SP, Skrinjar O, Binkley CM, Powell DK, Mattingly AC, Epstein FH, Fornwalt BK: **Quantification of left ventricular volumes, mass, and ejection fraction using cine displacement encoding with stimulated echoes (DENSE) MRI**. *J Magn Reson Imaging* 2014, **40**:398–406.

2. Yezzi Jr. AJ, Prince JL: **An Eulerian PDE approach for computing tissue thickness**. *IEEE Trans Med Imaging* 2003, **22**:1332–1339.

3. Zhong X, Spottiswoode BS, Meyer CH, Kramer CM, Epstein FH: **Imaging three-dimensional myocardial mechanics using navigator-gated volumetric spiral cine DENSE MRI.** *Magn Reson Med* 2010, **64**:1089–97.

4. Spottiswoode BS, Zhong X, Hess a T, Kramer CM, Meintjes EM, Mayosi BM, Epstein FH: **Tracking myocardial motion from cine DENSE images using spatiotemporal phase unwrapping and temporal fitting.** *IEEE Trans Med Imaging* 2007, **26**:15–30.

5. Zhong XD, Gibberman LB, Spottiswoode BS, Gilliam AD, Meyer CH, French BA, Epstein FH: **Comprehensive Cardiovascular magnetic resonance of myocardial mechanics in mice using three-dimensional cine DENSE**. *J Cardiovasc Magn Reson* 2011, **13**.

6. Helm RH, Leclercq C, Faris OP, Ozturk C, McVeigh E, Lardo AC, Kass DA: **Cardiac dyssynchrony analysis using circumferential versus longitudinal strain: implications for assessing cardiac resynchronization**. *Circulation* 2005, **111**:2760–2767.

Table S1. Magnetic Resonance Imaging Acquisition Parameters

	SSFP	DENSE (6 / 18 spirals)	TMF	T1-Weighted
Repetition time (ms)	3.16–3.37	17	12.3	700
Echo time (ms)	1.3–1.5	1.08	3.26	27
Field of view (mm^2)	[292–400] x[340–400]	360x360 / 340x340	240x320	373x460
Image matrix	[208–256] x256	128x128 / 214x214	192x256	208x256
Reconstructed pixel resolution (mm^2)	[1.3–1.4] x [1.3–1.4]	2.8x2.8 / 1.6x1.6	1.25x1.25	1.8x1.8
Acquisition Matrix	[146–256] x256	128x128 / 214x214	192x256	208x256
Slice thickness (mm)	8	8	5	8
Flip angle (°)	50	20, variable	30	180
Averages	1	1	3	1
True Temporal Resolution (ms)	33.9–50.6	34	36.9	700
Reconstructed Temporal Resolution (ms)	16.4–49.9	34	4.5–9.4	

SSFP: steady state free-precession; DENSE: displacement encoding with stimulated echoes; TMF: transmitral flow

Table S2. Coefficients of Variation, 95% Limits of Agreement, and Biases

	Inter-observer (n = 10)			Inter-test (n=9)		
**Cardiac Geometry**	**COV%**	**Limits**	**Bias**	**COV%**	**Limits**	**Bias**
LV mass (g)	4	-12, 13	0.4	5	-19, 9	-5.0
LV end systolic volume (mL)	11	-6, 20	7	6	-11, 8	-1.5
LV end diastolic volume (mL)	1	-5, 7	0.7	3	-17, 15	-0.8
Relative wall thickness (dimensionless)	7	-0.07, 0.08	0.01	5	-0.04, 0.05	0.006
Average LV thickness (mm)	4	-0.8, 0.6	-0.06	5	-1, 0.4	-0.3
Maximum LV thickness (mm)	6	-2.2, 1.7	-0.2	3	-0.7, 1.0	0.11
LV thickness at 90th percentile (mm)	4	-1.1, 1.0	-0.05	4	-1.1, 0.6	-0.3
**Cardiac Function**	**COV%**	**Limits**	**Bias**	**COV%**	**Limits**	**Bias**
LV ejection fraction (%)	7	-15, 5	-5	4	-7, 9	1.0
E/A (dimensionless)	7	-0.6, 0.4	-0.1	13	-1.5, 1.1	-0.2
E/E'(dimensionless)	28	-13, 9	-2	23	-3, 6	2
**Peak Strain**	**COV%**	**Limits**	**Bias**	**COV%**	**Limits**	**Bias**
Circumferential (%)	5	-2.4, -0.4	-1.4	5	-3.2, 2.7	-0.2
Radial (%)	13	-8, 15	-3	12	-10, 19	-4.5
Longitudinal (%)	10	-5.2, 5.5	0.2	12	-5, 5	-0.2
**Peak Systolic Strain Rate**	**COV%**	**Limits**	**Bias**	**COV%**	**Limits**	**Bias**
Circumferential (s^{-1})	4	-0.02, 0.09	0.04	7	-0.23, 0.30	0.04
Radial (s^{-1})	18	-0.29, 0.78	0.25	15	-0.93, 0.91	-0.01
Longitudinal (s^{-1})	34	-1.07, 0.54	-0.26	20	-0.58, 0.43	-0.07
**Peak Diastolic Strain Rate**	**COV%**	**Limits**	**Bias**	**COV%**	**Limits**	**Bias**
Circumferential (s^{-1})	5	-0.18, 0.07	-0.06	12	-0.29, 0.46	0.08
Radial (s^{-1})	12	-1.02, 0.57	-0.23	26	-1.78, 1.00	-0.39
Longitudinal (s^{-1})	35	-0.84, 1.55	0.36	12	-0.28, 0.35	0.04
**Cardiac Tosion/Synchrony**	**COV%**	**Limits**	**Bias**	**COV%**	**Limits**	**Bias**
Torsion (degree/cm)	4	-0.6, 0.2	-0.2	8	-1.1, 0.8	-0.2
CURE (dimensionless)	1	-0.03, 0.03	0.003	2	-0.07, 0.09	0.01
RURE (dimensionless)	4	-0.15, 0.16	-0.004	7	-0.18, 0.23	0.02
